# Can Plantar Pressure Distribution During Gait Be Estimated from Quiet Stance in Healthy Individuals?

**DOI:** 10.3390/jfmk10030301

**Published:** 2025-08-05

**Authors:** Marta Mirando, Chiara Pavese, Valeria Pingue, Stefania Sozzi, Antonio Nardone

**Affiliations:** 1 Department of Clinical-Surgical, Diagnostic and Pediatric Sciences, University of Pavia, 27100 Pavia, Italy; marta.mirando01@universitadipavia.it (M.M.); chiara.pavese@unipv.it (C.P.);; 2 Istituti Clinici Scientifici Maugeri IRCCS, Centro Studi Attività Motorie (CSAM) and Neurorehabilitation and Spinal Units of Pavia Institute, 27100 Pavia, Italy; 3 Department of Electrical, Computer and Biomedical Engineering, University of Pavia, 27100 Pavia, Italy; stefania.sozzi@unipv.it

**Keywords:** plantar pressures, quiet stance, gait, baropodometry, stabilometry, healthy subjects, rehabilitation

## Abstract

**Objectives**: We assessed the difference between quiet stance and gait in the spatial distribution and intensity of foot plantar pressures and whether it is possible to estimate the distribution during gait from data obtained during stance. **Methods**: A total of 60 healthy subjects with a mean age of 31.0 ± 9.4 years performed two trials for quiet stance and four trials for gait on a baropodometric walkway with their eyes open. Foot plantar pressures were recorded from 10 areas of the foot sole. **Results**: During quiet stance, the highest plantar pressure occurred at metatarsal heads (M2 to M4) and the medial (MH) and lateral halves of the heel (LH). During gait, the profile of plantar pressure values was like that during stance, but significantly higher. The differences concentrated at the big toe (T1), M2 to M4, MH, and LH, whilst toes (T2,3,4,5) and midfoot (MF) showed the smallest difference. A significant positive correlation was found between the corresponding areas of foot pressure during gait and stance. **Conclusions**: During quiet stance and gait, the overall profile of plantar pressure distribution was similar. During quiet stance, the subjects loaded more on the heels, in keeping with the known position of the center of pressure just in front of the ankles. During gait, higher pressures on the metatarsal areas are related to the forward propulsion of the center of mass. The correlation between the corresponding areas of foot pressure during gait and stance suggests that the pressure distribution during gait can partly be estimated from that during stance. This finding might be useful in most clinical settings when a single sensorized platform rather than a complete walkway is available.

## 1. Introduction

In assessing human gait and posture, foot pressures play a role in accessing the information hidden beneath the foot’s interaction with the surface in contact [1]. During quiet stance and gait, the spatial distribution of foot plantar pressures over time determines the average position of the center of pressure (CoP) as the centroid of the total number of active sensors [2,3]. During quiet stance, the CoP sways in both anteroposterior and mediolateral directions due to the forces exerted on the ground through the feet to control the center of mass (CoM) to maintain stability [4]. The sway of the CoP in both the AP and ML directions is a manifestation of the dynamic interplay between the gravitational forces acting on the CoM and the neuromuscular responses required to counteract these forces [5]. On the other hand, during the stance phase of gait, the progression of the CoP consists of a path formed by a series of coordinates passing from the hindfoot to the midfoot and finally to the forefoot, again as a product of the spatial and temporal distribution of the foot plantar pressures [2].

Analyzing plantar pressures during gait is essential for understanding foot function, biomechanics, and their implications for various activities of daily living and clinical conditions. By evaluating plantar pressure distribution, clinicians can identify asymmetries, abnormal loading patterns, and areas of excessive pressure, which may indicate underlying musculoskeletal or neurological impairments [6]. Initially, the analysis of foot pressures was used mostly for clinical foot deformities or foot illness diagnosis [7], such as those of patients affected by diabetes mellitus [8,9], calcaneal [10,11], or ankle fractures (see Mirando et al. [12] for a review) and pes cavus [13,14]. In these conditions, plantar pressures allow for the estimation of the severity of the pathology and assess the possible risk of complications [15].

Although plantar pressures during gait have been thoroughly studied in both healthy subjects and patients, little consideration has been given to plantar pressures during quiet stance [16]. For example, at variance with the latter condition, when the plantar pressures are constantly acting at the interface between the foot sole and the ground, during the stance phase of gait, the plantar pressures progress during three distinct phases (loading response, mid-stance, and terminal stance) [17,18]. In addition, the plantar pressures are affected by gait speed in a non-uniform way but with some differences in the various areas of the foot sole [19]. Finally, both leg and intrinsic foot muscles are active during double leg support [20,21] as much as during quiet stance, but they are active to a larger extent during the single leg stance phase of gait [20], thus affecting the intensity and pattern of distribution of foot plantar pressures [22].

To our knowledge, few data have been published directly comparing plantar pressures across different areas of the foot sole during balance and gait in the same healthy subjects. Dragulinescu et al. [22] reviewed the pattern of contact areas of the foot sole during different types of standing and gait tasks using different wearable devices. In turn, Cleland et al. [2] focused on the relationship between the contact area of the foot sole and the position of the CoP. They found that the location of CoP is a consequence of different contact regions spread across the foot sole and that, during standing, a great part of the foot sole is in contact with the ground at any time. On the contrary, during gait, only a small part of the foot sole is in contact with the ground. However, neither study focused on the differences in distribution and height of plantar pressures within the various areas of the foot sole between quiet stance and gait. Only Merry et al. [23] addressed this issue. They investigated which regions of the foot can differentiate walking, standing, and sitting in a small sample of young healthy subjects. They aimed to identify the foot sole areas most affected during each activity in a typical working environment to find a connection between work exposure and plantar heel pain.

Although quantitative differences exist in the distribution of plantar pressures between quiet standing and walking, it is not clear to what extent it is possible to estimate plantar pressure distribution during gait from data obtained during quiet stance. This finding might be of some interest since, in most clinical settings, a single sensorized platform rather than a complete walkway is available, thus allowing for recording plantar pressures during quiet stance but not during gait. In addition, we wondered whether any difference in foot plantar pressures between stance and gait was only related to the absolute values being higher during gait [23] or whether there were also spatial differences in plantar pressure distribution between the two tasks. These spatial differences could be the consequence of the biomechanical events characterizing quiet stance and gait and the larger activation of the intrinsic and extrinsic foot muscles under the latter condition [20]. Preliminary results have been presented in abstract form [24].

## 2. Materials and Methods

### 2.1. Subjects

This study was approved (#2461CE, 06 October 2020) by the local Ethics Committee of the Istituti Clinici Scientifici Maugeri of Pavia, Italy. Written informed consent was obtained from each subject before the experiment in accordance with the Declaration of Helsinki. The sample size of 60 was an estimate based on what is usual in the field [25] and considering the aim of measuring a diverse age group of healthy volunteers.

The inclusion criteria were the following: (1) age between 18 and 65 years old; (2) no cardiac, pulmonary, or neurological diseases; (3) no previous major trauma or surgical intervention on the musculoskeletal system; and (4) ability to understand and sign the informed consent form. The anthropometric measures collected in our sample were weight, height, and lower limb length (from the greater trochanter to the ground). The subjects were tested at the Centre for the Study of Motor Activities (CSAM) of the Istituti Clinici Scientifici Maugeri in Pavia.

### 2.2. Assessment Protocol

Plantar pressure was recorded using a baropodometric walkway (P-walk, BTS Bioengineering, Garbagnate Milanese, Italy). The multiple pressure platform system consists of four modules for a total of 9216 resistive load cells (2304 for each platform), with a total length of 2 m and a sampling frequency of 50 Hz. The walkway was calibrated using the manufacturer’s proprietary software. Calibration followed standard procedures, applying known weights and verifying sensor response. Prior validation studies regarding the above walkway support excellent accuracy (Pearson’s r = 0.99) and low intra-session variability (CV < 5%) during static loading tests [26,27].

Each subject performed two trials barefoot in the quiet stance condition with their eyes open (EO). The participants remained upright with the arms extended alongside the trunk, with the feet forming an open angle of 30° and the malleoli spaced 5 cm apart, following the international standardization criteria for baropodometric tests [28]. To ensure uniformity of position between the subjects, a foot template was placed on the first module of the platform, the one used for static measurements. The path length and surface of the 95% confidence ellipse of the center of pressure (CoP) were recorded for 30 s. The recording did not start immediately, but we waited about ten seconds to avoid recording any initial adaptation of the subjects. The mean of the two trials was calculated for the subsequent analyses.

Four gait trials with EO were performed, asking subjects to start when they felt ready at least two meters before the beginning of the platform, to walk at their usual speed, and to stop two meters after the end of the sensorized platform. We recorded walking speed and single and double support time from the mean of the four trials.

### 2.3. Plantar Pressure Evaluation

**Figure 1:**
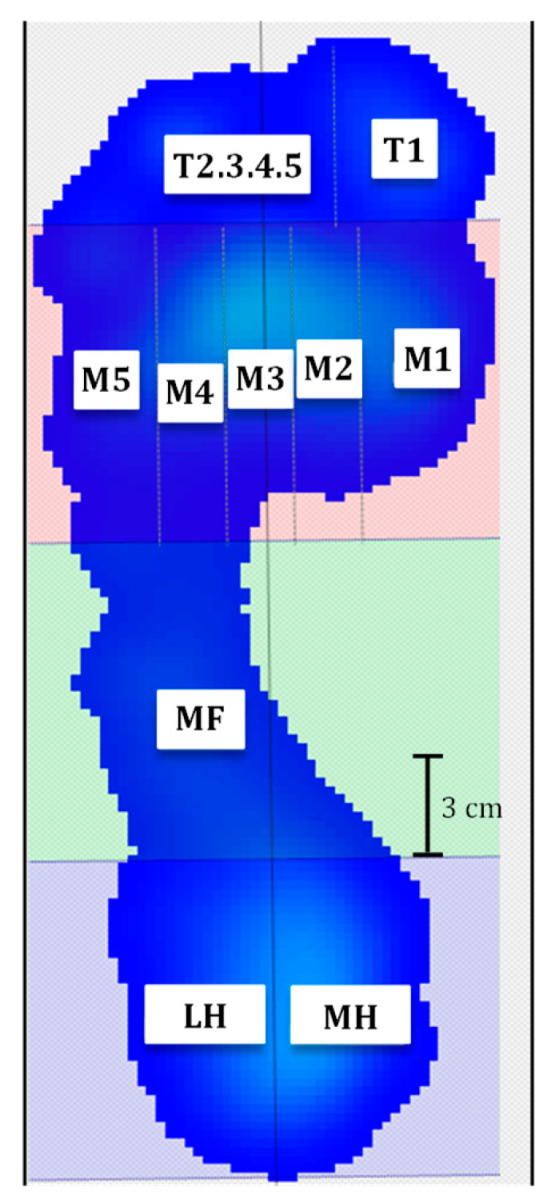
Graphical representation of foot areas in the left foot. The foot is divided into ten areas: T1, big toe; T2,3,4,5, toes 2 to 5; M1, metatarsal 1; M2, metatarsal 2; M3, metatarsal 3; M4, metatarsal 4; M5, metatarsal 5; MF, midfoot; MH, medial half of heel; and LH, lateral half of heel.

Under both static and walking condition, the software of the sensorized platform divided the foot into the following ten anatomical areas (Figure 1): T1, big toe; T2,3,4,5, toes 2 to 5; M1, metatarsal 1; M2, metatarsal 2; M3, metatarsal 3; M4, metatarsal 4; M5, metatarsal 5; MF, midfoot; MH, medial half of heel; and LH, lateral half of heel. For each area, peak plantar pressure (kPa) was calculated during both quiet stance and gait. The mean peak plantar pressure of the ten-foot areas was calculated separately from the trials under the static condition and the trials under the walking condition for each subject. In such a way, we further analyzed the average value of the areas of the right and the left footprints, which was carried out separately for the static and dynamic conditions.

In the case of gait trials, given the 2 m length of the walkway, one of the authors (M.M.) selected one step of the right foot and one of the left foot for each trial. Those footprints partially contained by the walkway or with unclearly delineated foot shape were excluded from further analysis. In the latter case, the trial was repeated to obtain four steps for each foot.

### 2.4. Statistical Analysis

The distribution of the data was assessed for skewness, kurtosis, and equality of variance (Levene’s test). As the assumption of normality was met for each variable, data were then expressed as the mean ± standard deviation (SD). For both the right and left foot, we first calculated the average of the peak plantar pressure of the ten-foot areas and tested whether the average value differed between the right and left foot (paired Student’s *t*-test). This was performed separately for quiet stance and gait. As we found no difference between the right and left foot mean pressure values during either quiet stance [26] or gait [29], for each condition, we considered the mean pressure of each corresponding area averaged over the two feet for further analysis.

We then assessed the difference in the ten plantar pressure areas and task (stance or gait) using a 2-way repeated measure analysis of variance (ANOVA) with quiet stance and gait and the ten areas as repeated measures. On the other hand, the differences in plantar pressure at each single-foot area calculated between gait and stance were analyzed using a one-way ANOVA. When the assumption of sphericity was violated, the Greenhouse–Geisser correction was applied. Partial eta squared (η^2^_p_) was reported as a measure of effect size, with 0.01, 0.06, and 0.14 considered as small, medium, and large effect sizes, respectively. Multiple comparison of the mean differences was performed using Tukey’s adjustment. The Cohen’s d effect size (with 0.2, 0.5, and 0.8 considered as small, medium, and large effect sizes, respectively) was also reported. *p* values < 0.05 were considered statistically significant.

The relationships between plantar pressures during gait and age, weight, height, body mass index (BMI), and plantar pressures during stance were investigated using regression analyses. All statistical tests were performed using SPSS version 21 for Windows (IBM Corporation, Armonk, NY, USA).

## 3. Results

### 3.1. Subject Characteristics

We enrolled 60 healthy subjects, including 29 men (48.3%) and 31 women (51.7%), with a mean age of 31.0 ± 9.4 years (standard deviation, SD), weight of 67.0 ± 13.7 kg, height of 172.0 ± 8.9 cm, body mass index of 22.5 ± 3.1 kg/m^2^, and lower limb length of 90.2 ± 5.9 cm and 90.2 ± 5.8 cm for the right and left limbs, respectively.

### 3.2. CoP Sway and Total Plantar Pressure Distribution During Quiet Stance

During quiet stance, the surface of the 95% confidence ellipse of the CoP and its path were 36.8 ± 12.9 mm^2^ and 128.3 ± 29.8 mm, respectively. The average peak pressure between the ten areas of the right and left foot was 30.0 ± 6.6 kPa and 32.5 ± 6.9 kPa (paired *t*-test, *p* = 0.10, d = 1.05), respectively. As no differences were found between the right and left foot, the mean pressure of each corresponding area from both feet was considered for further analysis.

### 3.3. Spatial-Temporal Variables of Gait and Total Plantar Pressure Distribution

During gait, the mean speed was 1.20 ± 0.16 m/s. The mean time of single support was 0.46 ± 0.05 s for the right foot and 0.44 ± 0.08 s for the left foot. The mean time of double support was 0.10 ± 0.03 s for the right foot and 0.11 ± 0.03 s for the left foot. We did not find any difference in the support times between the two feet (paired *t*-test for the time of single and double support, respectively; *p* = 0.20, d = 0.17 and *p* = 0.55, d = 0.08).

The mean peak plantar pressure of the ten areas of the right and left foot was 132.0 ± 12.5 kPa and 129.1 ± 11.2 kPa, respectively. As in the quiet stance condition, also during gait, the peak plantar pressure averaged over the ten plantar areas did not show a significant (paired *t*-test, *p* = 0.11, d = 1.17) difference between the right and left foot. Therefore, also in this case, the mean peak plantar pressure of each corresponding area from both feet was considered for further analysis.

**Figure 2:**
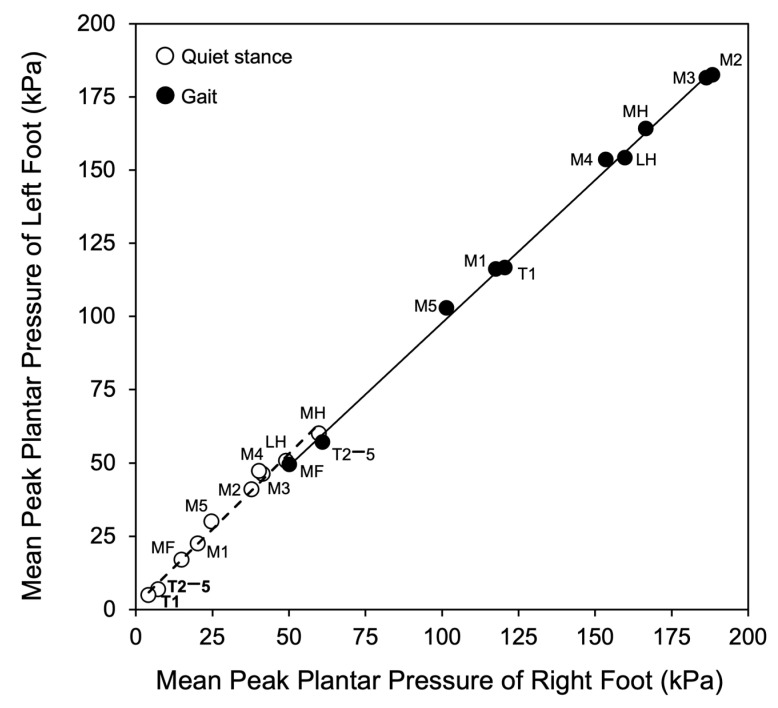
Relationship between the mean peak plantar pressure of each anatomical area of the foot sole during quiet stance and gait in the right and left foot. Each symbol represents the mean peak plantar pressure of the corresponding area of both feet of all subjects.

The strong similarity between corresponding areas of the right and left foot is apparent in Figure 2, showing that both in the case of quiet stance and gait a significant (*p* < 0.0001) positive relationship (stance, y = 1.03x + 1.54, R^2^ = 0.99; gait, y = 0.97x + 0.47, R^2^ = 0.99) existed between mean peak plantar pressures averaged over each of the ten-foot areas of right and left foot.

### 3.4. Peak Pressures of the Foot Areas During Quiet Stance and Gait

**Figure 3:**
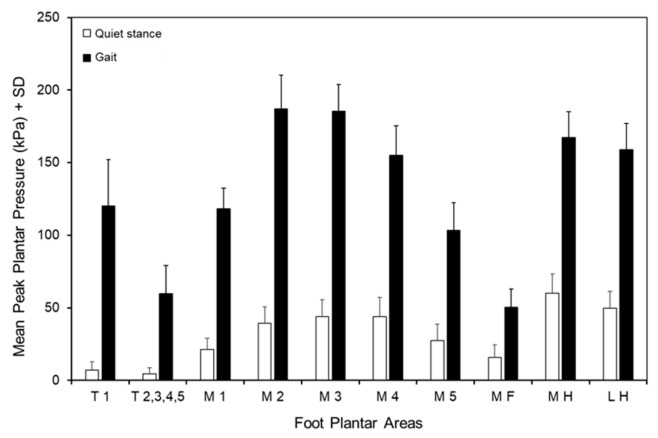
Mean + standard deviation (SD) of peak plantar pressure distribution over the ten anatomical areas of the foot sole during quiet stance (open bars) and gait (filled bars). T1, big toe; T2,3,4,5, toes 2 to 5; M1, metatarsal 1; M2, metatarsal 2; M3, metatarsal 3; M4, metatarsal 4; M5, metatarsal 5; MF, midfoot; MH, medial half of heel; LH, lateral half of heel.

As shown in Figure 3, there was a significant difference between quiet stance and gait condition (F (1,59) = 2345.93, *p* < 0.001, Ƞ^2^_p_ = 0.98). There was a significant difference between foot areas (F (4.35,256.99) = 500.51, *p* < 0.001, Ƞ^2^_p_ = 0.89) and a significant interaction between quiet stance and gait condition and foot areas (F (3.98,234.71) = 207.58, *p* < 0.001, Ƞ^2^_p_ = 0.78). As shown in Figure 3 and Table 1, during quiet stance (open bars), the highest mean peak pressures were recorded at the metatarsal heads (from M2 to M4) and the heel (MH and LH). These areas showed significantly (*p* < 0.001 and d > 0.6 for all comparisons) higher pressure than the others. In turn, the pressure at the metatarsal areas M2 and M3 was significantly (*p* < 0.05 and d > 0.33 for all comparisons) lower than at the heel (MH and LH). The lowest pressures occurred at the toes (T1 and T2–T5) and at the midfoot (MF) (*p* < 0.001 and d > 0.5 for all comparisons).

During gait (Figure 3 and Table 1), overall, the profile of the plantar pressure distribution was superimposable to that during quiet stance. Plantar pressures were significantly (*p* < 0.001) higher in all areas of the foot sole than the corresponding ones during quiet stance. As in the latter case, the highest pressures were found at the metatarsal heads (from M2 to M4) and at the heel (both MH and LH) compared with other areas (*p* < 0.001 and d > 3.04 for all comparisons). At variance with a quiet stance, the pressure at the metatarsal areas M2 and M3 was significantly (*p* < 0.001 and d > 1.04 for all comparisons) higher than at the heel (MH and LH). On the contrary, the lowest pressure occurred at the toes (T2–T5, *p* < 0.001 and d > 2.4 for all comparisons), except for the big toe (T1), which showed a great increase in pressure with respect to T2–T5 (*p* < 0.001, d = 6.37), and at the midfoot (MF, *p* < 0.001, d > 2.5 for all comparisons). There was no difference in the pressure value between T2–T5 and MF area (*p* = 0.07, d = 0.52).

### 3.5. Differences in Peak Pressures of the Foot Areas Between Gait and Quiet Stance

**Figure 4:**
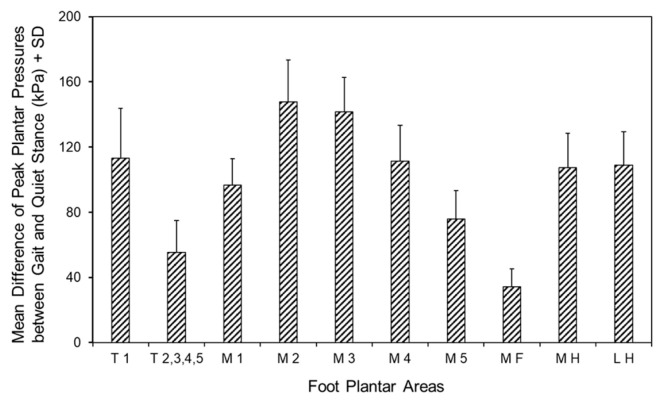
Mean + SD of the difference in peak plantar pressure distribution between quiet stance and gait in the ten anatomical areas of the foot sole, averaged from both feet. T1, big toe; T2,3,4,5, toes 2 to 5; M1, metatarsal 1; M2, metatarsal 2; M3, metatarsal 3; M4, metatarsal 4; M5, metatarsal 5; MF, midfoot; MH, medial half of the heel; LH, lateral half of the heel.

To put into evidence the changes in plantar pressure distribution between quiet stance and gait, we calculated the difference in pressure at each relevant foot area (Figure 4). As already observed, there was a significant difference between the ten-foot area (F (3.86,227.97) = 219.03, *p* < 0.001, Ƞ^2^_p_ = 0.79). It appeared that the difference in pressure between gait and stance was not evenly distributed but concentrated at the big toe (T1), metatarsal heads (especially, M2 and M3), and heel (MH and LH); these areas showed a significantly (*p* < 0.001, d > 1.3) higher difference than the others. Toes 2 to 5 (T2,3,4,5), fifth metatarsal head (M5), and the midfoot (MF) showed the smallest difference between gait and stance of all areas (*p* < 0.001, d > 0.98 for all comparisons).

### 3.6. Relationships Between Quiet Stance, Gait, and BMI

**Figure 5:**
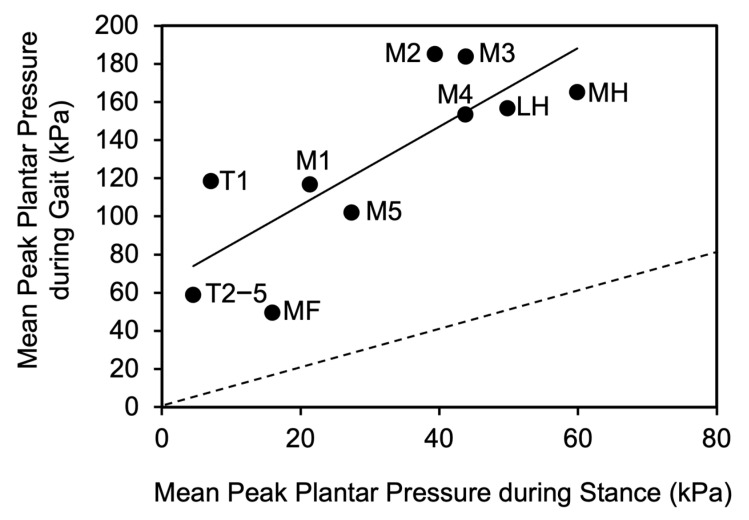
Relationship between the peak plantar pressure of the ten anatomical areas of the foot sole averaged from both feet during gait and quiet stance. T1, big toe; T2,3,4,5, toes 2 to 5; M1, metatarsal 1; M2, metatarsal 2; M3, metatarsal 3; M4, metatarsal 4; M5, metatarsal 5; MF, midfoot; MH, medial half of the heel; LH, lateral half of the heel. The dashed line indicates the identity line.

When the average peak pressures of the ten areas of the foot sole during gait were correlated with the corresponding pressures during quiet stance, a significant positive relationship (y = 2.1x + 64.7, *p* < 0.01, R^2^ = 0.64) (Figure 5) was found. On the contrary, no relationship was found between the average peak pressure during gait or stance and the following variables: BMI, gait speed, time of single and double contact, and surface of the 95% confidence ellipse of the CoP and its path (see Table S1).

## 4. Discussion

In this study, we found that, despite the expected quantitative differences in mean plantar pressures between foot areas and between quiet stance and gait, the distribution of plantar pressures during gait is only slightly qualitatively different compared to quiet stance. During the latter condition, the regions of greatest pressure correspond to the heel and the metatarsals, from the second to the fourth. Conversely, during gait, the areas of greatest pressure correspond to the big toe and, in a similar way to quiet stance, to the heel and the metatarsals. Interestingly, a strong relationship was observed between the peak pressures at corresponding areas of the foot during stance and gait.

### 4.1. Plantar Pressures During Quiet Stance

In static conditions, such as quiet standing, the distribution of pressure across the foot is relatively uniform and stable. Static standing involves minimal movement and predominantly vertical forces acting on the foot. Under this condition, the body weight is distributed across both feet, primarily through the heels and the balls of the feet, but more on the heels. This phenomenon could be related to the known projection of the center of pressure (CoP) just in front of the ankle [30]. In fact, during quiet stance, the distance between the internal malleolus and the anterior–posterior position of the CoP is on average less than 6 cm, corresponding to about 24% of foot length. As suggested by Hennig and Sterzing [31] and Machado et al. [32], the midfoot may play a key role in balance control since it seems to be the most sensitive region to somatosensory stimuli. In this context, Fernández-Seguín et al. [14] have shown that during quiet stance, the areas where the greatest pressure is exerted are precisely at the level of the forefoot, second, and third metatarsal heads, as in our case. This distribution could be explained by the conformation of the foot, with the bones of the second and third metatarsal heads getting stuck between the wedge-shaped joints and therefore having less freedom of movement. As a result, they are less able to distribute the load they receive [33].

### 4.2. Plantar Pressures During Gait

As shown by Merry et al. [23], in comparing static and dynamic foot support, the overall pressure distribution appears similar in both conditions, with higher pressures at the heel and forefoot regions. During gait, the dynamic nature of the task leads to constantly changing pressure distribution across the foot [2]. Indeed, smooth progression of the body over the supporting foot during the stance phase is achieved by the actions of three functional rockers: the heel, the ankle, and the forefoot [17,18]. During foot transitions through the rocker phases, pressure shifts and concentrates in specific areas. In the heel strike phase (MH, LH), initial contact generates high pressure. During the ankle rocker phase, pressure moves toward the midfoot (MF), facilitating dorsiflexion and propulsion. In the forefoot rocker phase (M2, M3, M4), pressure concentrates under the metatarsal heads, enabling push-off (T1) [17]. Consequently, the temporal characteristics of pressure change significantly, and these differences in pressure distribution between static and dynamic conditions can be attributed to the functional demands of gait [34]. In fact, during walking, the foot must act as a flexible structure that absorbs shock, adapts to uneven terrain, and generates propulsive forces. The three rocker phases represent a coordinated sequence of movements that optimize these functions. This dynamic loading pattern helps distribute the forces evenly across the foot, reducing the risk of localized pressure-related injuries [35].

### 4.3. Relationship Between Quiet Stance and Gait

The above considerations explain why the regression between the corresponding areas of foot pressure during gait and quiet stance in healthy subjects accounts for 64% of the variance of plantar pressures during gait that can be predicted by quiet stance. Therefore, it is possible to obtain a rather good estimate of foot pressure distribution during gait from its distribution during quiet stance. Overall, our results make plantar pressure collection more accessible to clinical practice, also in terms of the cost and time of evaluations and analyses. In fact, many studies have only analyzed plantar pressures under static conditions, as the dynamic analysis requires the availability of a sensorized walkway [14,36,37]. The relationship between mean plantar pressure in corresponding foot sole areas during gait and quiet stance is stronger when data points are closer to the regression line. A close alignment with the regression line suggests a well-defined, predictable relationship between pressure points, allowing for an accurate assessment of pressure distribution across the foot, enabling healthcare professionals to identify high-risk areas with greater confidence. Understanding these foot support areas is crucial for identifying patients prone to ulcers or other complications. It also facilitates the development of personalized interventions, such as insoles or orthopedic shoes, to enhance support and reduce injury risk. Additionally, monitoring plantar pressure helps evaluate treatment effectiveness in improving foot support and preventing complications [38].

### 4.4. Comparison with Literature Findings

In addition to these insights, our study offers a novel and clinically meaningful advancement over the existing literature. Specifically, Merry et al. (2020) [23] investigated plantar pressure characteristics to distinguish among sitting, standing, and walking. Their aim was primarily categorical classification of postures in work environments, especially concerning plantar heel pain. In contrast, our objective was to analyze whether gait pressure profiles could be inferred from static measurements in the same individuals, providing a continuous and predictive biomechanical relationship. Furthermore, our study involved a substantially large sample size to evaluate spatial and quantitative differences. Although the overall pattern of plantar pressure distribution in our study was similar to that reported by [23], one important point of divergence between the two studies lies in the absolute pressure values reported. In our study, peak plantar pressures during gait reached values well over 180 kPa in some forefoot areas (e.g., M2 and M3), while Merry et al. (2020) [23] reported substantially lower regional peak pressures. This discrepancy can be attributed to multiple methodological factors. For example, we used a resistive sensor walkway with high resolution and analyzed a well-defined cohort of 60 healthy adults, whereas Merry et al. (2020) [23] used a different system with a smaller sample size. In addition, variations in protocol, such as walking both along linear and curvilinear trajectories, barefoot versus shod conditions, or trial repetitions, may further contribute to inconsistencies in reported pressure values. These methodological differences highlight the importance of standardization in plantar pressure studies. However, it should also be noted that, while absolute values may differ between studies, similar conclusions can still be drawn.

Although several studies suggest that the peak pressure increases linearly with an increase in walking speed [19,39,40], we did not find a correlation between walking speed and plantar pressures or between plantar pressures and the sway area or path during quiet stance. The lack of correlation between gait speed and plantar pressures may be related to the different methodologies used in our study compared to others. For example, in the study by Hughes et al. [19], subjects had to walk at three different speeds, whereas in our study, subjects walked at their preferred speed. In the latter condition, we found a very small variation coefficient (13.3%) of the walking speed, which explains the small scatter of the data and presumably prevented the finding of a significant correlation between plantar pressures and walking speed.

We did not find a relationship between BMI and peak plantar pressures, either in the quiet stance or during gait. This is not surprising, since the current literature is unclear as to whether there is a relationship between peak plantar pressure and BMI during standing. For example, Lalande et al. [41] found a relationship between peak plantar pressures and subjects’ weight as well as BMI during quiet stance. On the other hand, Arnold et al. [42] found a positive relationship between increasing body mass and peak and mean plantar pressure variables for most foot areas, but the relationship was highly dependent on the area of interest of the foot. However, the correlation between body mass and plantar pressure only accounted for 16% of the variance in peak plantar pressure. In addition, the same authors deliberately increased the body mass of their subjects up to 15 kg, whereas in our study, the body mass was not varied, thus preventing a sufficient scatter of data to highlight a possible linear relationship between pressure and BMI. Finally, Phetean et al. [43] found a weak degree of association between body weight, BMI, and plantar pressures. Overall, such differences could be explained by differences in the methodology used in the different studies.

### 4.5. Limitations

The results of this study should be considered in light of the following limitations. Firstly, due to the lack of comparable data in the literature, it was not possible to perform an a priori sample size calculation. However, the number of subjects in our study was comparable to other studies of gait and foot pressure [44]. Secondly, the number of steps required to characterize plantar pressure during gait was small (four), but this is a matter of debate in the literature [45]. In clinical analysis of gait [46], three steps are commonly used, and three to five steps are thought to be sufficient to record plantar pressure in adults aged 20 to 35 years [47]. As Kernozek et al. [48] reported for peak pressure, a minimum of two to three footfalls was needed to reach a reliability coefficient of 0.70, indicating good reliability, regardless of the foot area considered. In addition, the plantar pressure distribution obtained in our study was similar to that found in the literature, with a larger number of footprints [49] and a smaller number of subjects [23]. For example, Putti et al. [50] found that two to three steps were sufficient to capture consistent pressure values with excellent intra-subject repeatability. These studies suggest that key pressure parameters remain stable and reproducible with as few as two steps, especially when consistent walking protocols are followed.

Finally, comparison of our data with other normative references for plantar pressures is complicated by a lack of agreement on how to divide the foot and how to examine the areas under the foot. Despite the different ways of splitting the foot areas, our findings for plantar pressures are consistent with the literature [41,51,52]. Various approaches have been used, including expressing pressure as an absolute measure, or scaled to peak plantar pressure, foot length, body mass, or body mass index for ten, nine, five, or three areas of the foot [50,53,54]. Despite this variable approach, only small associations between body weight, body mass index, and plantar pressure data have been reported in the literature, suggesting that scaling to these parameters is not advantageous [43].

## 5. Conclusions

In summary, the present study investigated the distribution of plantar pressures in a sample of young healthy adults in two different conditions, quiet stance and gait. The results showed that the highest peak pressure was in the heel during stance, rather than in the metatarsal region and the greater toe, as occurred during gait. However, the overall similar profile of the plantar pressure distribution under static and dynamic conditions was reflected by a significant positive correlation between the corresponding areas of foot pressure during gait and those during quiet stance. This finding allows the pressure distribution during gait to be estimated to a rather good extent from its distribution during quiet stance, but, since these results are collected from healthy subjects, they need to be confirmed in a cohort of patients to be applied in a clinical setting.

The assessment of plantar pressures in healthy individuals during both standing and walking goes beyond traditional pathological studies to explore the natural biomechanics of the foot. By directly comparing static and dynamic conditions in the same subjects, this approach reveals how the foot adapts to movement, revealing important pressure shifts that might be overlooked.

## Figures and Tables

**Table 1:** Mean ± standard deviation of peak plantar pressure distribution values over the ten anatomical areas of the foot sole averaged from both feet during quiet stance and gait.

Foot Areas	Peak Pressure (kPa) During Quiet Stance	Peak Pressure (kPa) During Gait
T1	7.1 ± 5.8	118.6 ± 34.2
T2–5	4.5 ± 4.0	59.0 ± 20.2
M1	21.3 ± 7.7	116.8 ± 17.6
M2	39.3 ± 11.3	185.4 ± 26.6
M3	43.8 ± 11.8	183.9 ± 22.4
M4	43.7 ± 13.2	153.6 ± 23.1
M5	27.4 ± 11.3	102.2 ± 21.0
MF	15.9 ± 8.5	49.7 ± 13.5
MH	59.9 ± 13.5	165.3 ± 23.2
LH	49.8 ± 11.4	157.0 ± 23.3

## Data Availability

The mean data presented in this study are available on request from the corresponding author.

## Supplementary Materials

The following supporting information can be downloaded at https://www.mdpi.com/article/10.3390/jfmk10030301/s1. Table S1: Linear relationships between the average value of the ten areas of the right and the left foot sole during gait or stance and BMI, gait speed, time of single and double contact, and surface of the 95% confidence ellipse of the CoP and its path.

## Abbreviations

The following abbreviations are used in this manuscript:

CoP	Center of pressure
CoM	Center of mass
EO	Eyes open
kPa	Kilopascal
T1	Big toe
T2,3,4,5	Toes 2 to 5
M1	Metatarsal 1
M2	Metatarsal 2
M3	Metatarsal 3
M4	Metatarsal 4
M5	Metatarsal 5
MF	Midfoot
MH	Medial half of the heel
LH	Lateral half of the heel
SD	Standard deviation
ANOVA	Analysis of variance
BMI	Body mass index
