# Supplementary material for: Can Plantar Pressure Distribution During Gait Be Estimated from Quiet Stance in Healthy Individuals?
Source: J Funct Morphol Kinesiol. 2025 Aug 5;10(3):301. doi: 10.3390/jfmk10030301 (PMC12372002; doi:10.3390/jfmk10030301)
Supplement: Supplementary file 1 [file jfmk-10-00301-s001.zip › jfmk-3757078-supplementary.pdf]

Table S1. Linear relationships between the average value of the ten areas of the right and the left footsole during gait or stance and BMI, gait speed, time of single and double contact, surface of the 95% confidence ellipse of the CoP, and its path. For each pair, the linear regression equation, R^2, and p-value are reported.

Independent Variable	Dependent Variable	Equation	R^2	p-value
BMI	Footsole Pressure during Stance	y = 0.069x + 20.3	0.022	0.26
Gait speed		y = 0.001x + 1.2	0.003	0.70
Time of Single Contact		y = 0.001x + 0.4	0.026	0.22
Time of Double Contact		y = 0.001x + 0.1	0.031	0.18
95% confidence ellipse of the CoP		y = 0.008x + 26.1	0.011	0.50
CoP path		y = 0.290x + 142.6	0.001	0.88
BMI	Footsole Pressure during Gait	y = 0.012x + 20.9	0.002	0.77
Gait Speed		y = 0.002x + 0.9	0.021	0.26
Time of Single Contact		y = -0.001x + 0.6	0.02	0.28
Time of Double Contact		y = -0.001x + 0.2	0.047	0.10
95% confidence ellipse of the CoP		y = 0.002x +129.2	0.0002	0.95
CoP path		y = -0.044x + 135.9	0.030	0.44

BMI, Body Mass Index; CoP, Centre of Pressure.
